# Spatio-temporal transcriptome and storage compound profiles of developing faba bean (*Vicia faba*) seed tissues

**DOI:** 10.3389/fpls.2024.1284997

**Published:** 2024-02-06

**Authors:** Hannah Ohm, Ganapathi Varma Saripella, Per Hofvander, Åsa Grimberg

**Affiliations:** Department of Plant Breeding, Swedish University of Agricultural Sciences (SLU), Lomma, Sweden

**Keywords:** *Vicia faba*, seed development, seed storage proteins (SSP), RNA-sequencing (RNA-seq), transcription factor (TF)

## Abstract

Faba bean (*Vicia faba*) is a legume grown in diverse climate zones with a high potential for increased cultivation and use in food due to its nutritional seeds. In this study, we characterized seed tissue development in faba bean to identify key developmental processes; from embryo expansion at the expense of the endosperm to the maturing storage stages of the bean seed. A spatio-temporal transcriptome profiling analysis, combined with chemical nutrient analysis of protein, starch, and lipid, of endosperm and embryo tissues at different developmental stages, revealed gene expression patterns, transcriptional networks, and biochemical pathways in faba bean. We identified key players in the LAFL (LEC1, ABI3, FUS3, and LEC2) transcription factor network as well as their major repressors VAL1 and ASIL1. Our results showed that proteins accumulated not only in the embryo but also in the endosperm. Starch accumulated throughout seed development and oil content increased during seed development but at very low levels. The patterns of differentially expressed transcripts encoding proteins with functions in the corresponding metabolic pathways for the synthesis of these storage compounds, to a high extent, aligned with these findings. However, the early expression of transcripts encoding WRI1 combined with the late expression of oil body proteins indicated a not manifested high potential for lipid biosynthesis and oil storage. Altogether, this study contributes to increased knowledge regarding seed developmental processes applicable to future breeding methods and seed quality improvement for faba bean.

## 1 Introduction

*Vicia faba* has several common names, including broad bean, horse bean, field bean, fava bean, and faba bean. It is an economically minor crop globally, with production quantities making out less than 2% of that of soybean which is the dominating grain legume today (FAOSTAT, 2021). Originally domesticated in North-West Syria around 10,000 BP, the faba bean is regarded as one of the oldest domesticated crops (O’Sullivan and Angra, 2016) with no identified wild ancestor. The main consumable is the fresh or dried seeds of faba bean that, due to their high protein content (25%-33% of dry-matter basis) (Heuzé et al., 2021) and balanced amino acid profile, are valuable as part of a healthy human diet as well as for animal feedstock (Ellwood et al., 2008; Multari et al., 2015). Increased cultivation of legumes such as faba bean, and their extended usage for human food consumption can support the ongoing dietary shift from animal to plant-based proteins (Röös et al., 2020). In addition, increased cultivation would contribute to a more sustainable food production system since legumes show low greenhouse gas- and water footprints (Semba et al., 2021) and have the ability to fertilize the soil by fixing atmospheric nitrogen, leading to a significant reduction of inputs in the agricultural sector (Lybæk and Hauggaard-Nielsen, 2019). Due to adaptation to diverse climate zones, faba bean also bears the potential to aid our transition to greater self-sufficiency of plant protein (Multari et al., 2015). Thus, it is a crop of nutritional and ecological value providing various ecosystem services (Punia et al., 2019), and can give an increased economic value if used to a higher extent in food products. Nevertheless, the biological processes behind nutrient storage and the regulatory network of gene expression during seed development of faba bean are still poorly investigated, the first paths being paved by Borisjuk et al. (1995; 2002). Indeed, knowledge about the genetic traits defining seed composition of stored macromolecules is fundamental for directed breeding and other approaches toward improved seed quality and quantity (Kang et al., 2016).

Generally, seed development can be split into the three phases histodifferentiation, seed filling, and desiccation. They span from the embryo’s initial development and cell division to the exhaustion of the endosperm, and the accumulation of storage compounds in the cotyledons, until final seed desiccation and dormancy (Weber et al., 2005). The metabolic and regulatory pathways involved in these processes are well described for legume model species *Medicago truncatula* and *Lotus japonica*, as well as for other economically important legumes, such as soybean (*Glycine max*) and pea (*Pisum sativum*) (Gallardo et al., 2007; Dam et al., 2009; Malovichko et al., 2020; Sun et al., 2020). There is, however, a great variation in storage strategies within the legume family (Song et al., 2017), which motivates the need for research focusing on faba bean seed development. Due to the very large genome of the faba bean (13 Gb), with, until very recently, no reference genome available (Jayakodi et al., 2023), a transcriptomic approach of an RNA-sequencing (RNA-seq) analysis constituted an accessible method to study the genetic networks. This technique provides snapshots of gene expression in tissue and is a powerful tool for comparisons of differentially expressed transcripts (DETs), given certain circumstances such as treatments, varieties, or developmental stages.

The focus of this study was the major nutrient storage compartment of the faba bean seed, the embryo, and the tissue that surrounds it at an early developmental stage, the endosperm. For this we used two varieties that are commonly grown in Europe, one with white flower color (var. Taifun) and one with variegated flower color (var. Fanfare). Because of the absence of tannins in the seed coat, white flower colored faba bean types are often preferred for use in monogastric feed (Gutierrez et al., 2020), however yields are often lower than varieties with variegated flower color (Halling & Hagman, 2020). Our study did not place emphasis on pericarp tissue and variety variations in tannins because it is already known that genes encoding transcription factors (TFs) involved in the anthocyanin biosynthesis pathway control the presence of tannins in seed coat and flowers (Gutierrez et al., 2020).

Here, we combined transcriptome profiling with chemical analyses of storage compounds, to elucidate the major developmental stages for faba bean seed development and investigated the composition of key storage compounds in the embryo and endosperm tissues separately. This study elucidated the major metabolic shifts during seed filling of faba bean, with distinct phases of parallel processes occurring in the different tissues of the developing seed. To the best of our knowledge, it is the first of its kind to shed light on the transcriptional regulatory network underlying storage compound accumulation in this species.

## 2 Materials and methods

### 2.1 Plant material and growth conditions

Two commercially available faba bean varieties (var.), Taifun and Fanfare, with white and variegated flower colors respectively (Norddeutsche Pflanzenzucht, Germany), were cultivated in soil (50% peat, pH 5.5–6.5, added per m^3^ soil: 5.5 kg lime, NPK 11-5-18 kg, 200 g micronutrients and 100 g iron) under greenhouse (for RNA-seq analysis) or Biotron (for metabolite analysis) conditions: 18°C –21°C light 6–22 h < 200 W/m^2^, with a weekly supplement of Ca and NPK nutrient solution. Top shoots of plants were removed after a couple of weeks of growth to stimulate vegetative-reproductive competition and enhance flower retention for increased pod setting. Seeds were sampled at different developmental stages and different tissues (endosperm, embryo, pericarp, petals, and sepals) were isolated using a scalpel, spoon, and forceps. The sampled tissues were then snap-frozen in liquid nitrogen until stored at -80°C. Sampling was done continuously for 2–3 months after sowing until enough plant material of each developmental stage had been accessed, and sampling was done at approx. the same time point of the day to avoid any potential bias in gene expression due to circadian rhythm.

### 2.2 Tissue fixation and staining for microscopy

Longitudinal- and cross-sections of faba bean seeds at different developmental stages were fixed in 4% paraformaldehyde in Sorensens Phosphate Buffer (0.1M Na-phosphate, pH 7.0), shaken overnight, and rinsed in the same buffer the next day. Samples were stored in fresh phosphate buffer at 4°C until dehydrated in a series of EtOH concentrations: 30% for 3 h, 50% overnight, 70% for 3h, 95% for 3 h, and 100% EtOH for 24 h. Next, samples were cleared while shaking in a series of different EtOH-Xylene-solutions at different time periods: (2:1) overnight day one, (1:1) overnight day two, (0:1) two times for 3 h, and (0:1) for 24 h. Samples were embedded in melted paraffin (Histowax, Histolab, Gothenburg, Sweden) at 55°C for 3 x 24 h, cast into a mold, and stored dark at 4°C. Paraffin blocks were cut with Leitz Wetzlar 1515 rotary microtome (Leica Microsystems GmbH, Wetzlar, Germany) into 5–10 µm thin slices, and placed on fresh object glass (SuperFrost Plus, Menzel, Thermo Fisher Scientific, Waltham, Massachusetts, USA) at 37°C –40°C for 24–48 h, then stored at room temperature.

De-paraffinized samples were stained with Lugol’s iodine (L) solution for starch detection, Light green (LG) for protein detection, and Sudan black B (SB) for lipid detection (Grimberg et al., 2020), and mounted with cover glass using Pertex mounting solution (Histolab, Gothenburg, Sweden). Microscopy images were acquired using a light microscope (Leica, DFC 450C camera, Wetzlar, Germany).

### 2.3 Determination of lipid, protein, and starch content

Seeds were sampled at various developmental stages, and embryo, endosperm, and pericarp were isolated, weighed, and freeze-dried for 48 h at 2.5 mbar and -60°C (Edwards Modulyo, UK). To obtain dry matter content, freeze-dried material was weighed before being stored at -80°C. Subsequent compound analysis was made on samples representing both varieties on four developmental stages (I-IV), with three biological replicates each. One biological replicate consisted of tissue pooled from 1–3 individual plants which were unique for that replicate.

For lipid extraction, 40 mg dry weight (dw) from each sample was extracted according to Bligh and Dyer (1959). An aliquot of 1 mL was concentrated in a glass tube on hot sand under nitrogen gas to a suitable volume and then applied on silica 60 thin layer chromatography plates of size 20 cm x 20 cm (Merck, Darmstadt, Germany) to separate lipid classes in heptane:diethyl ether:acetic acid (70:30:1 by vol.) by developing the plate in room temperature to its full length. After drying the plate and spraying it with primuline, free fatty acids (FFA) and triacylglycerol (TAG) species were located under UV light according to authentic standards. Identified regions of silica were scraped off and fatty acids in lipids were methylated into fatty acid methyl esters with 2% (v/v) sulphuric acid in dry methanol. Gas chromatography analysis of fatty acid methyl esters was performed on a CP-wax 58 column (FFAP-CB, 50 m, 0.32 mm inner diameter, 0.20 µm film, Varian, Palo Alto, USA) using an Agilent Technologies 8860 gas chromatograph (Santa Clara, US). Fatty acid methyl esters were quantified by using heptadecanoic acid methyl ester (Larodan, Solna, Sweden) as internal standard.

For protein and starch analyses, the freeze-dried material was first ground into fine flour in a mixer mill (MM 400, Retsch GmbH, Haan, Germany) at 30 Hz, using glass beads. For protein content determination, calculated from the total N residue, 3 mg flour was analyzed for total N quantification with the elemental analyzer (Flash 2000 Elemental Analyzer, Thermo Scientific). We followed the protocol for “Determination of total carbon and total nitrogen by dry combustion method (Dumas method) using a CN elemental analyzer” (Bieganowski et al., 2015), with Alfalfa as the reference standard (Krotz and Galotta, 2020). Subsequently, the 6.25 conversion factor converted total N content to protein content.

Total starch content was determined enzymatically on 40 mg flour with the Megazyme Total Starch HK Assay kit, K-TSHK (Megazyme, Brey, Ireland). Free sugars were first removed from flour by extraction with ethanol washes, followed by enzymatic degradation of starch according to the manufacturer’s protocol, except for a down-scale of the colorimetric analysis step for the determination of glucose from degraded starch to fit a 96-well plate format. The absorbance was measured at 510 nm on a microplate spectrophotometer (Multiskan GO, ThermoFisher Scientific, MA, USA).

### 2.4 Sample preparation and RNA isolation

While frozen, plant tissues were ground in steel containers with 9–12 mm Ø steel beads chilled in N_2_ to a fine powder using a mixer mill (MM 400, Retsch GmbH, Haan, Germany) at 30 Hz. RNA extraction was done following the protocol of RNeasy Mini Kit (Qiagen, Hilden, Germany), using the buffer RLC together with DTT. RNA extraction was done on endosperm and embryo tissue, representing both varieties in three developmental stages (I–III), with two or three biological replicates each (**Supplementary Table 1**). Additionally, RNA was extracted from pericarp tissue (developmental stage II), and petals and sepals for use in *de-novo* transcriptome assembly. RNA concentrations and RIN-values were measured with a NanoDrop (Thermo Fisher Scientific, Waltham, USA) and BioAnalyzer 2000 (Agilent Technologies, Santa Clara, USA). Samples were DNAse-treated with the TURBO DNA-free™ Kit (Invitrogen, MA, USA) before being sent for RNA sequencing.

### 2.5 RNA sequencing and quality control

Paired-end sequence reads were generated using Illumina high-throughput sequencing by Eurofins Genomics (Ebersberg, Germany), and initial Quality Control (QC) check was performed. Removal of ribosomal RNAs was done by aligning reads with SILVA (Quast et al., 2013) and Rfam (Kalvari et al., 2021) databases using Sortmerna-v2.1b (Kopylova et al., 2012), followed by TruSeq3 adapter trimming with Trimmomatic-v0.36 (Bolger et al., 2014) setting MINLEN:20 in bases and SLIDINGWINDOW:5:20 along with other default parameters. The second round of QC checks was performed on independent samples with FastQC v0.11.7 (Andrews, 2010) and multiple sample visualization MultiQC v1.6 (Ewels et al., 2016).

### 2.6 *De-Novo* transcriptome assembly and functional annotation of differentially expressed transcripts

To perform *de-novo* transcriptome assembly, we used Trinity v3.2.2 workflow (Haas et al., 2013) and included all reads from all sequenced samples for the assembly. An additional SuperTranscripts script (https://github.com/trinityrnaseq/trinityrnaseq/wiki/SuperTranscripts) was applied to generate Trinity transcripts (Davidson et al., 2017). Transcript abundance was estimated using Salmon v1.3.0 (Patro et al., 2017). Raw read counts were used for Differential Expression analysis with DESeq2 (Anders and Huber, 2010; Anders et al., 2013) and in-built cross-sample “Relative Log Expression” (Love et al., 2014) normalization was performed by using minimum quality criteria. Further, two thresholds were set FDR < 0.05, and both FDR < 0.05 and Log2FoldChange (Log2FC) > 1.0, of which the most stringent was used for further downstream analysis. To annotate the function of expressed transcripts, the Trinity contig sequences were analyzed with blastx (NCBI-BLAST-v2.9.0+), parameters were set as -max_target_seqs 1, -evalue 1e^-5^ and searched against the Swiss-Prot/Uniprot database and scanned with TransDecoder-v5.5.0 (https://github.com/TransDecoder/TransDecoder) to predict open reading frames (ORFs). The predicted ORFs were further analyzed with the Trinotate-v3.2.2 pipeline (github.com/Trinotate/Trinotate.github.io). These ORFs were searched against Swiss-Prot/Uniprot database (uniprot_sprot) with blastp (-max_target_seqs 1, -e-value 1e^-5^) and then searched against other databases, such as, 1) Pfam-A.hmm release 34.0 using hmmscan v3.3, 2) TmHMM-2.0c, and 3) SignalP v.4.1. All the results were merged with Trinotate pipeline scripts, and an additional script (extract_GO_assignments_from_Trinotate_xls.pl) was used to generate Gene Ontology (GO) terms. Nucleotide sequences and corresponding ORFs of the generated transcripts were searched with blastx and blastp against predicted transcripts of three close relatives of faba bean, 1) *Medicago truncatula* (assembly: GCF_003473485.1), 2) *Pisum sativum* (Pea) (assembly: GCA_900700895.2) and 3) *Glycine max* (Soybean) (assembly: GCF_000004515.6). In addition plant model organism *Arabidopsis thaliana* (assembly: GCF_000001735.3) was included in comparative studies.

Plant-based TF databases such as PlantTFDB v5.0 (http://planttfdb.cbi.pku.edu.cn/) and iTAK v18.12 (Zheng et al., 2016) were included in the annotation. Database for plant model *Vicia faba* species is unavailable in both these TF databases, therefore other species such as *Medicago Truncatula*, *Glycine max*, and *Arabidopsis thaliana* databases were included for comparative analysis. Transcript expression levels were normalized with the Trimmed mean of M-values (TMM) method, due to the underlying distribution of expressed transcripts between samples being markedly different.

To highlight any sample variation we performed a principal component analysis (PCA) of the read count data. Quality filter was applied to transcripts and reduced to 41,821 by keeping transcripts that are expressed in at least two samples. Variance stabilizing transformation (VST) and plotPCA functions from the DESeq2 package where applied (Anders and Huber, 2010; Anders et al., 2013).

### 2.7 KEGG and GO enrichment analysis

The Kyoto Encyclopedia of Genes and Genomes (KEGG) enrichment analysis was performed on the list of genes identified to be differentially expressed in all pairwise genotype comparisons, by using obtained gene coordinates from three model species *Arabidopsis thaliana*, *Medicago Truncatula*, and *Glycine max*. The KEGG Pathway enrichment analysis was performed with Gene Set Enrichment Analysis (GSEA) of KEGG, tested with submodule gseKEGG from the clusterProfiler (v3.18.1) R-package (Wu et al., 2021), with settings nPerm = 10,000, value of p cutoff = 0.01 and keeping remaining settings as default. The Gene Ontology (GO) over-representation test was performed using *Arabidopsis thaliana* coordinates, with enrichGO submodule from the clusterProfiler R-package (Yu et al., 2012) with settings pvalueCutoff = 0.05, qvalueCutoff = 0.05 and keeping other settings as default.

All scripts used in this study are available under: https://github.com/gvarmaslu/RNAseq_Faba-bean.

### 2.8 Statistical tests

Descriptive statistics were used to describe the characteristics of different seed tissue and developmental stages. Statistical analysis was performed on data of DETs and nutrient analyses by comparing means of development stages and tissues for each var. separately by a one-way analysis of variance (ANOVA) to test the significance of differences among samples followed by a posthoc Tukey’s test at significance level p ≤ 0.05 (Minitab 21.3.1, State College, PA, US). Means that do not share a letter in graphs are significantly different. Standard deviations (s.d) are indicated in graphs with error bars.

## 3 Results

### 3.1 Definition of seed tissue developmental stages

**Figure 1:**
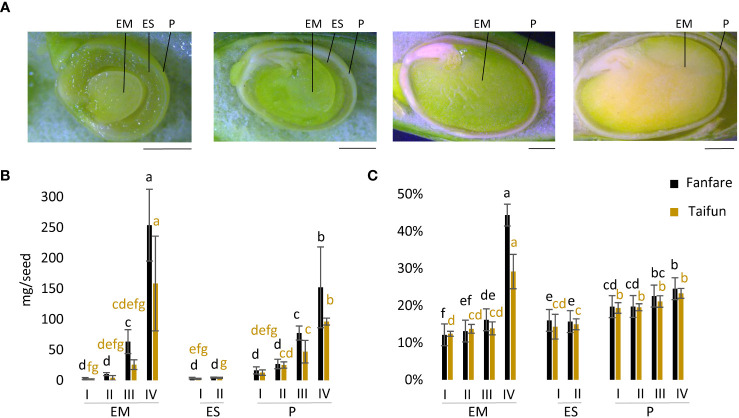
Microscopy images and dry matter content of faba bean seed developmental stages. **(A)** Microscopy images of longitudinal cut-open faba bean seeds at developmental stages I, II, III, and IV (from left to right). Scale bar: 2.5 mm. Dry weight of seeds in **(B)** mg/seed and **(C)** as % dry matter of embryo (EM), endosperm (ES), and pericarp (P) in var. Fanfare and Taifun at different developmental stages. Results are the mean ± s.d from three biological replicates. Means that do not share a letter are significantly different according to Tukey’s test (p < 0.05), comparisons are made for each variety (color indicated) separately.

To identify different tissues in faba bean seeds, we performed a visual examination of beans that were dissected horizontally at three different developmental stages. Guided by previous descriptions of faba bean seed development (Patrick and Stoddard, 2010) we identified four distinct developmental stages, I to IV, used in this study (**Figure 1A**). These stages were determined based on visual assessment, according to the proportions of different seed tissues, i.e. of the endosperm and embryo. At stage I at ~15 days after flowering (DaF), the earliest phase investigated here, the embryo was small but clearly visible, and surrounded by a relatively thick layer of endosperm. At stage II at ~17 DaF, the embryo was already occupying most of the seed space, surrounded by only a thin layer of endosperm. At stage III at ~32 DaF the seed had entered a desiccation phase and the color of the pericarp had changed from green to yellow-green, but with the embryo still being green. At this point, the bean was almost completely filled with cotyledons/embryo and the pericarp (seed coat) had stiffened. The final stage IV at ~45 DaF displayed a desiccating seed, and now also with the embryo/cotyledons shifting from green to yellow. In line with these visual observations, the size of the embryo and pericarp (mg dry matter per seed) increased during seed development, but not that of the endosperm (**Figure 1B**). There was a trend of larger embryo and pericarp size of the var. Fanfare as compared to var. Taifun at the later developmental stages (**Figure 1B**). Stage IV clearly comprised the desiccating, mature, and larger seed with significantly higher dw content of the embryo than its preceding immature stages (**Figure 1C**).

**Figure 2:**
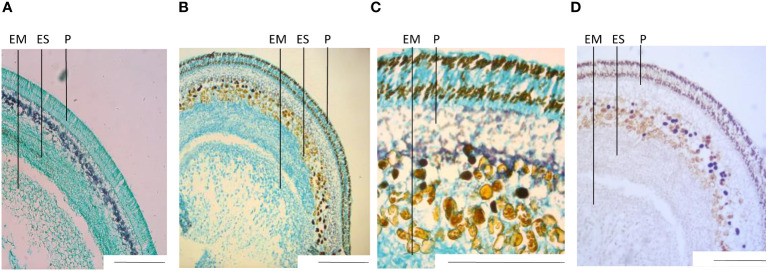
Transversal sections of fixed seeds, histochemically stained. Staining was done with Lugol’s iodine solution (L) for starch detection, light green (LG) for protein recognition, and Sudan Black (SB) for lipid visualization. **(A)** var. Taifun and **(B)** var. Fanfare double stained with L+LG. **(C)** Close-up of Fanfare, double stained with L+LG. **(D)** Fanfare stained with SB. All images are from seed developmental stage I Scale bar: 2 mm. EM= embryo, ES= endosperm, P= pericarp.

Histochemical staining and microscopy imaging of sections of fixed and paraffin-embedded seed parts served as additional anatomical references and as a confirmation of our definition of developmental stages. The outermost layers of the seed make out the seed coat, with the turquoise coloring of the light green (LG) staining, indicating protein-rich tissue and including the purple layer of the Lugol (L) staining, indicating a starch-rich section (**Figures 2A–C**). Going inward, the following layers of endosperm and embryo both showed to be protein-rich, with no or very little starch visible when double-stained in LG and L. Sudan black (SB) staining indicated low levels of lipids in the outer parts of the seed coat, and very low levels in the embryo (**Figure 2D**).

When comparing images of stained sections (**Figure 2**) with unstained sections (**Supplementary Figures 1A–C**), we noticed small dark-brown compartments present in the unstained sections of var. Fanfare that were not present in var. Taifun. Fanfare is a variety with variegated flowers that contain tannins in the seeds, which most likely explain the presence of these dark-brown substances since they could not be observed in sections from the white-flowering and tannin-free variety Taifun. The dark-brown compartments were present in two independent layers in the seed coat, and as part of cell compartments in the outer endosperm tissue of the faba bean, defining the border between (and including) the endosperm and seed coat. Tannic cell walls have been detected at the outer surface of the endosperm in *Arabidopsis thaliana* (Demonsais et al., 2020), and shown to concentrate in the central vacuole early postfertilization and subsequently oxidize, lending a brown hue to the seed coat in the seeds of *Arabidopsis thaliana* (Haughn and Chaudhury, 2005).

### 3.2 Characterization of seed starch, protein, and oil composition

To elucidate the nutrient storage strategy of the developing faba bean seed, storage compound levels were determined in the embryo at the four different developmental stages, and in the endosperm at the two earliest developmental stages (later stages had almost no visible endosperm tissue).

**Figure 3:**
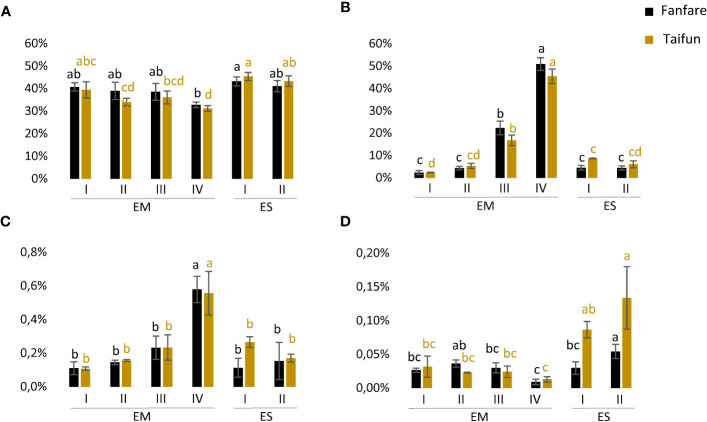
Accumulation of storage compounds in developing faba bean seeds. **(A)** Protein content (% by dw), **(B)** starch content (% by dw), **(C)** TAG content (% by dw), and **(D)** FFA content (% by dw) in faba bean embryo (EM) and endosperm (ES) at developmental stages I-IV. Results are the mean ± s.d from three biological replicates. Means that do not share a letter are significantly different according to Tukey’s test (p < 0.05), comparisons are made for each variety (color indicated) separately.

Protein levels were high and relatively stable and ranged from 31%–41% of the embryo dw, and were even higher, 41%–45%, of the endosperm dw, with a slightly decreasing trend in the embryo as the seed matured (**Figure 3A**). The opposite trend was shown for both starch and TAG (oil) levels, which were increasing for every developmental stage with clear peaks of doubled values in stage IV as compared to stage III (**Figures 3B, C**). Starch levels ranged from as low as 2% up to 51% of embryo dw during seed development, but remained at a relatively low and stable level of 5%–9% of the endosperm dw. The level of TAG ranged between 0.11%–0.58% of the embryo dw and 0.11%–0.27% of the endosperm dw. The levels of FFA ranged between 0.02%–0.04% of the embryo dw and 0.01%–0.13% of the endosperm dw (**Figure 3D**). Except for protein levels, the endosperm was showing generally lower contents of the analyzed storage compounds than the embryo. The pattern of nutrient accumulation was similar for the two varieties studied.

### 3.3 Sequencing data analysis and functional annotation of transcripts

To explore the transcriptional dynamics of faba bean seed development, high-throughput next-generation transcriptome sequencing with Illumina technology (RNA-seq) was performed on three developmental stages and two different tissues, which on average resulted in 76 million paired-end raw reads per sample. After the adaptor sequences and low-quality sequences (about 10-12%) reads were removed, on average 68 million clean reads per sample were obtained. In total 2,350 million reads were used for *de-novo* transcriptome assembly (**Supplementary Table 2**). The Q30 percentage was 94% and the GC (Guanine and Cytosine) content was 41.7%. A total of 227,336 genes were predicted from *de-novo* assembly out of which 59,505 *de-novo* transcripts were predicted with open reading frames (ORFs). The transcripts were annotated by blast searches against local databases of three legume species *Glycine max* (soybean), *Medicago truncatula* (barrel clover), and *Cicer arietinum* (chickpea), as well as the plant model species *Arabidopsis thaliana* (thale cress), data is shown in **Supplementary Table 3**. This resulted in an average of 28,221 unique annotated transcripts with ORFs, out of which 14,821 were in agreement between all model species. A principal component analysis (PCA) of the read count data confirmed consistent grouping of biological replicates based on their tissue types, with the exception of one endosperm replicate (**Supplementary Figure 2**). Further, the PCA revealed distinct clustering among the three different developmental stages of the embryo. In contrast, the two developmental stages of the endosperm did not exhibit clear grouping. Notably, the varieties could not be clearly distinguished from each other in this analysis, which indicates high similarities between them.

### 3.4 Identification of differentially expressed transcripts during *Vicia faba* seed development

**Figure 4:**
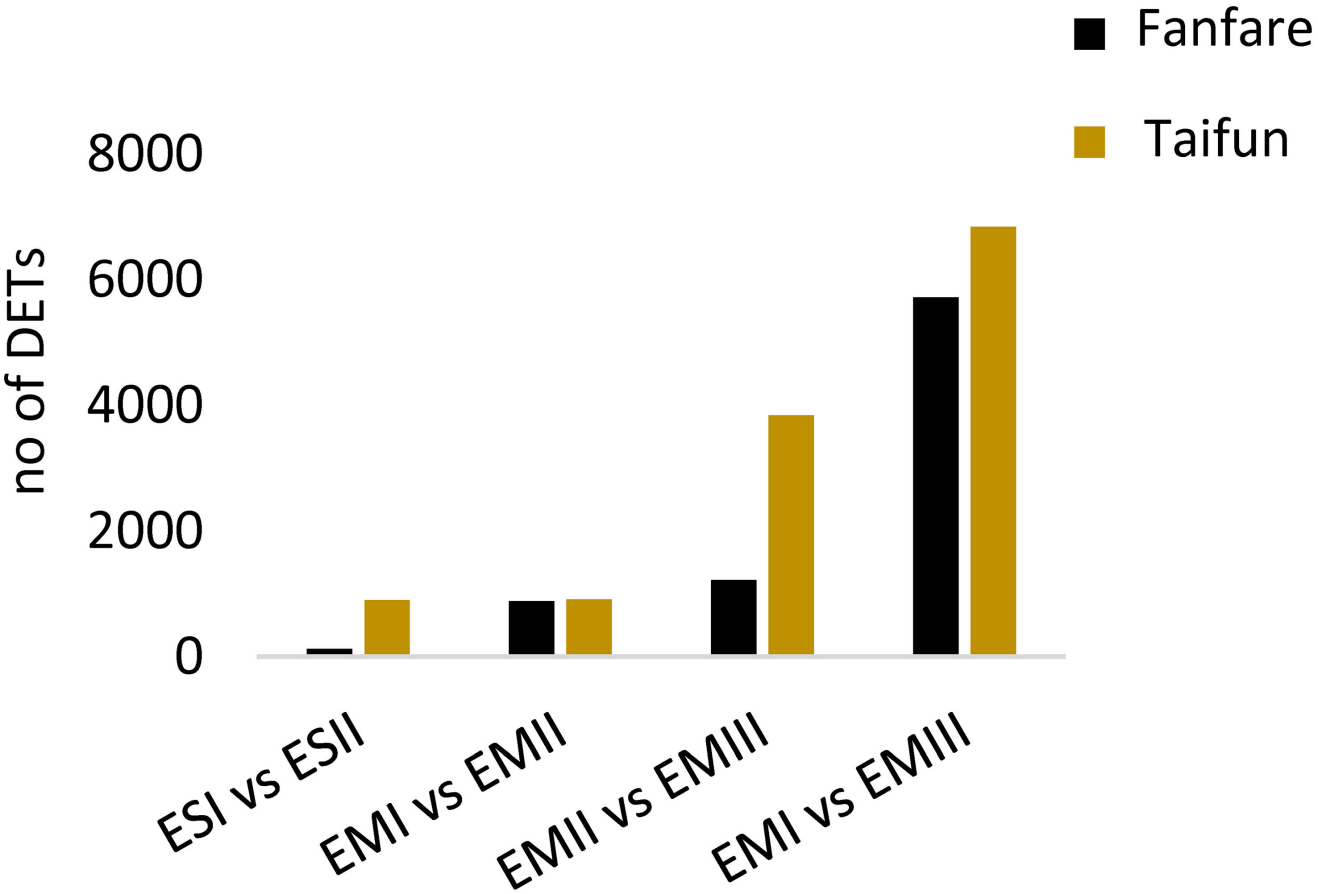
Differentially expressed transcripts in faba bean seeds. Comparison of the number of DETs between different developmental stages of seed tissue in faba bean var. Fanfare and Taifun. Further information on the pairwise comparisons at different thresholds is listed in **Supplementary Dataset 1**.

To get an overview of differences in gene expression between developmental stages of seed tissues, we determined the number of DETs in pairwise comparisons, based on the total 227,336 predicted genes (**Figure 4**; **Supplementary Table 4**; **Supplementary Dataset 1**). Generally, the biggest differences were found when comparing the latest with the earliest developmental stages, III vs I, in the embryo, for which around 6,000 transcripts were differentially expressed. A lower number of DETs were found between stage II vs I, than between stage III vs II. The number of DETs between endosperm tissue at stage II vs I was lower or equal to the number of DETs found in the embryo tissue at the same stages. DETs were also found between the white flowering var. Taifun and the variegated var. Fanfare, these differences were however not investigated further in this study. Instead, due to the high similarities found between the varieties in general, which are also seen in the abovementioned PCA (**Supplementary Figure 2**), the varieties are serving as biological replicates in this context.

### 3.5 Differentially expressed transcripts encoding transcription factors in developing seed tissues

**Figure 5:**
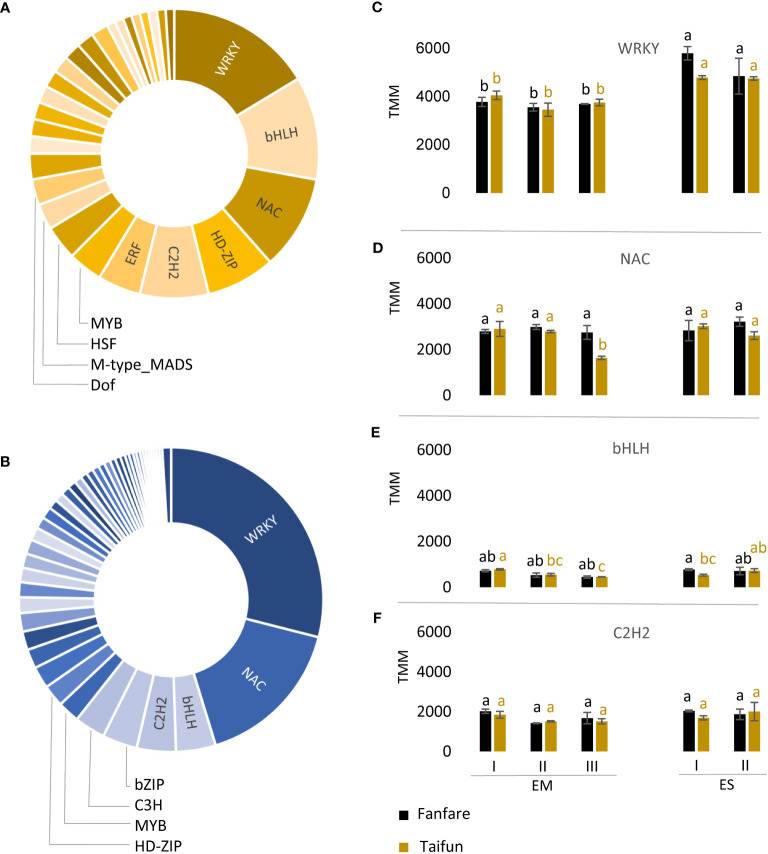
Transcription factor families shown to be differentially expressed in faba bean seeds during development. Pie charts of the distribution of DETs annotated to encode genes in TF families in **(A)** endosperm (yellow) and **(B)** embryo (blue) as an average between var. Fanfare and Taifun. **(C)** Expression levels for the DETs of the most common TF families in embryo (EM) and endosperm (ES) during the different developmental stages I to III were WRKY, **(D)** NAC, **(E)** bHLH, and **(F)** C2H2. Results are the mean ± s.d from three biological replicates. Means that do not share a letter are significantly different according to Tukey´s test (p < 0.05), comparisons are made for each variety (color indicated) separately.

To understand the regulatory mechanisms of seed development, we investigated the list of transcripts that showed a differential expression during embryo and endosperm development and were annotated as plant TFs (**Figure 5**). They accounted for 915 out of the 10,217 DETs identified from all pairwise comparisons of embryo and endosperm tissue, as an average of the two var. Fanfare and Taifun. Of these TF families, those represented in the list of DETs to the highest extent were WRKY, NAC, bHLH, and C2H2, for both embryo and endosperm (**Figures 5A, B**; **Supplementary Table 5**). The embryo tissue showed a much higher number of DETs belonging to TFs (n=858), than the endosperm tissue (n=59). The expression levels of the most common DETs encoding TFs showed substantial variation, with the highest expression for the WRKY family in both endosperm and embryo tissue (**Figure 5C**).

**Figure 6:**
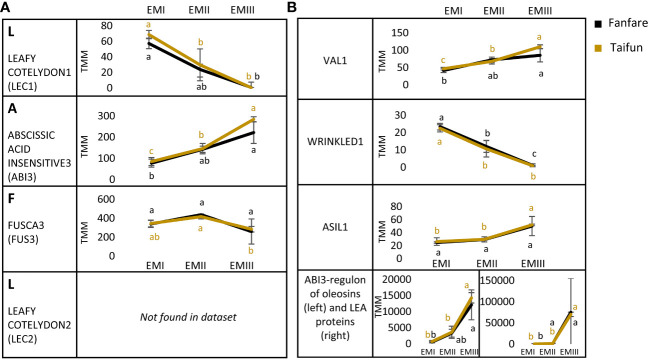
Differentially expressed transcripts (DETs) during faba bean embryo development, annotated to seed transcription factors (TFs). **(A)** Master regulator TFs, called the LAFL-network and **(B)** seed developmental transcription factor VAL1, fatty acid synthesis inducing transcription factor WRI1, ASIL1, putative ABI3-regulons part of the oleosin family and putative ABI3-regulons part of the late embryogenesis abundant (LEA) proteins. All expression levels are in TMM and show the var. Fanfare and Taifun. Results are the mean ± s.d of biological replicates. Means that do not share a letter are significantly different according to Tukey’s test (p < 0.05), comparisons are made for each variety (color indicated) separately.

To elucidate the connection between seed nutrient storage strategies and seed tissue development in faba bean, we looked further into transcripts annotated to specific TFs known to affect storage patterns in the embryo tissue. The LAFL network (LEAFY COTYLEDON1 (LEC1), ABSCISSIC ACID INSENSITIVE3 (ABI3), FUSCA3 (FUS3) and LEAFY COTYLEDON2 (LEC2)) is a set of TFs in plants that act as master regulators in seed development, triggering a cascade of secondary TFs, and regulating major hormone and signaling pathways (Lalanne et al., 2021). We found expressed transcripts annotated to encode three out of these four TFs in the developing faba bean embryo tissue (**Figure 6A**). Transcripts annotated to LEC1 showed decreasing expression with embryo development, while ABI3 was instead increasing. The expression level for the transcript annotated to FUS3 remained more or less constant throughout embryo development. Interestingly, transcripts homologous to LEC2 were not found in our dataset. Furthermore, transcripts annotated to other important TFs for embryo development or regulation of seed storage biosynthesis were looked at specifically (**Figure 6B**). The VIVIPAROUS1/ABI3-LIKE (VAL) TFs are known repressors of the LAFL network, enabling the transition from the embryonic to the vegetative state of the seedling (Jia et al., 2014). We found increasing expression of transcripts annotated as VAL1, but no DETs of VAL2, in the developing faba bean embryo. Transcripts annotated as the glycolysis and fatty acid synthesis regulating transcription factor WRINKLED1 (WRI1) were mainly expressed during the early stages of embryo development, with a significantly declining expression towards later stages. ASIL1 (for *Arabidopsis thaliana* 6b-interacting protein 1-like 1) contributes to the repression of the LAFL network by binding to its GT element (Gao et al., 2009). We found transcripts annotated as ASIL1 to remain at stable expression levels in the two earlier stages of embryo development, but increasing towards developmental stage three. Transcripts of the ABI3-regulon of putative oleosins and late embryogenesis abundant proteins showed a clear increasing expression during embryo development.

### 3.6 Seed storage strategies in developing embryo

**Figure 7:**
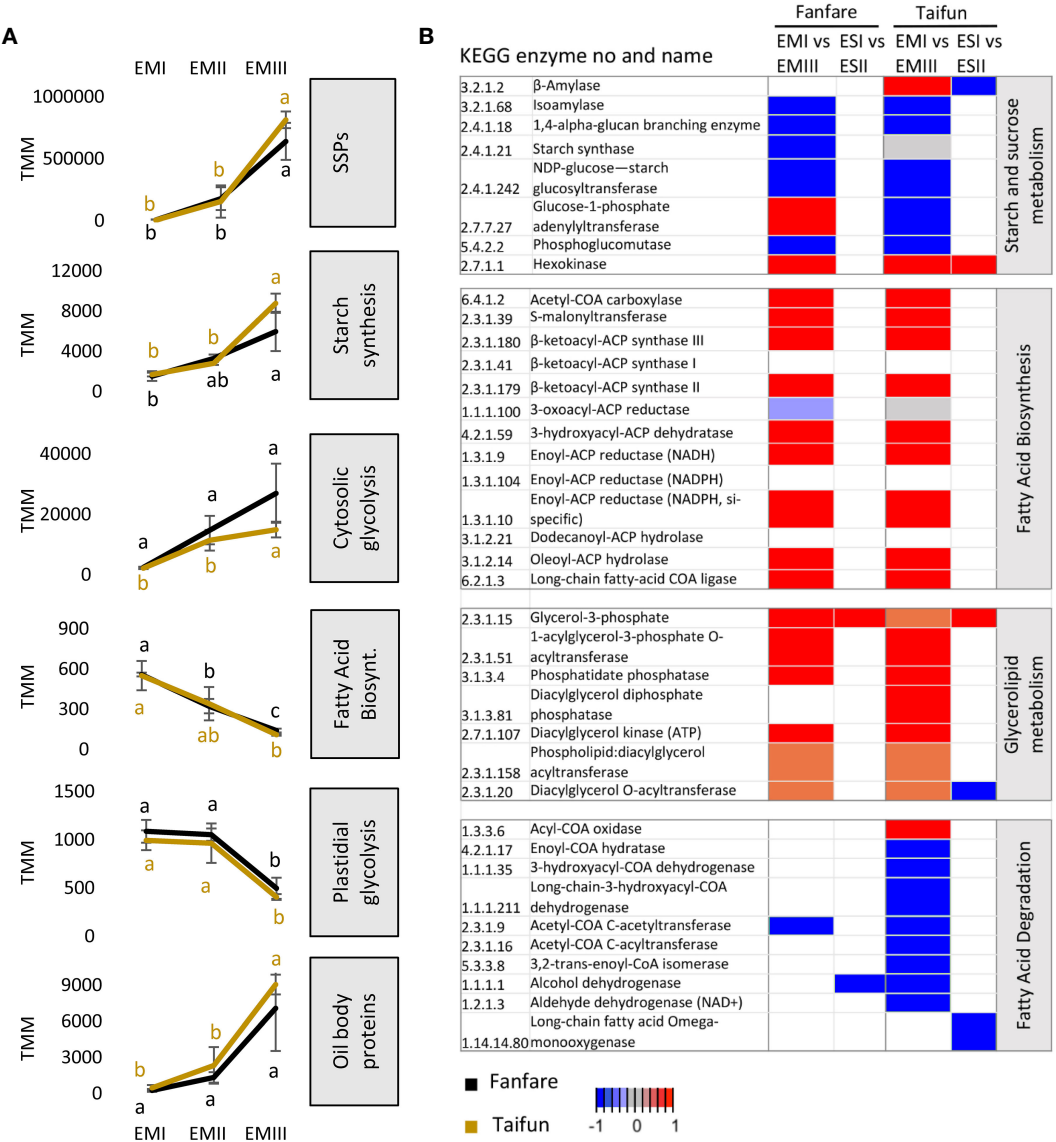
Differential expression of transcripts encoding proteins involved in major metabolic pathways of storage compounds. **(A)** Aggregated expression levels (TMM) of selected groups of differentially expressed transcripts during embryo development in the var. Fanfare and Taifun. Gene names and transcript IDs for the homologs used in the graphs are listed in **Supplementary Dataset 2**. **(B)** Heatmap of selected genes from enriched KEGG pathways of four major metabolic pathways for the biosynthesis of storage compounds. The expression pattern is shown as a relative color scheme for each row, comparing the different developmental stages of embryo and endosperm tissues in faba bean varieties Taifun and Fanfare. Red is indicating decreasing expression (higher expression at early compared to late developmental stage) and blue is indicating increasing expression (lower expression at early compared to late developmental stage). Results are the mean ± s.d of biological replicates. Means that do not share a letter are significantly different according to Tukey’s test (p < 0.05), comparisons are made for each variety (color indicated) separately.

To get an overview of the transcriptional changes during embryo and endosperm development that are involved in central carbon metabolism, we identified DETs of selected genes encoding functions in storage protein, starch, and oil synthesis pathways (**Figure 7A**). KEGG enrichment scores for the relevant molecular pathways are depicted as a heatmap where blue and red colors indicate negative and positive differential expression over the developmental stages in each tissue (**Figure 7B**). In general, the expression patterns of the transcripts annotated as enzymes in the biosynthesis pathways of the major storage compounds were very similar for the two different varieties studied. Large changes were instead observed between different developmental stages in the embryo and only small changes were occurring in the endosperm.

Transcripts annotated as the seed storage proteins cupin, legumin, globulin, and vicilin were all highly differentially expressed in the seed tissues, with an increased expression during embryo development (**Figure 7A**). For starch and sucrose metabolism, the expression of transcripts was in general increasing in the embryo during seed development (**Figure 7B**). However, for the hexokinase that catalyzes the conversion of glucose to G6P, as well as fructose to F6P, the transcript expression trend was the opposite. For beta-amylase and glucose-1-phosphate adenyltransferase, the transcript expression trends in the embryo were different for the two varieties. Furthermore, while the transcripts annotated as beta-amylase and hexokinase were differentially expressed in the endosperm in Taifun, none of them showed differential expression in Fanfare. For the initial cytosolic steps of the glycolytic pathway, which regulate the conversion of sucrose to hexose phosphates, the faba bean embryo showed increased transcript expression during seed development. For the final steps of the plastidic glycolytic pathway, in which pyruvate is converted to acetyl–coenzyme A (CoA), which is a substrate for fatty acid synthesis in the plastid, the aggregated expression of transcripts was significantly lower than in the cytosol and was instead decreasing during embryo development. In the final steps of the starch and sucrose metabolism, leading towards the formation of starch, the faba bean embryo showed increasing transcript expression during embryo development.

In the glycerolipid metabolism in plants, fatty acids are first synthesized in the chloroplasts and later, after being carried into the endoplasmic reticulum as acyl-CoA, ester-linked with a hydroxyl group of glycerol to form TAGs that are stored in oil bodies (Schmid, 2021). All major enzymatic steps of the fatty acid biosynthesis pathway, except one, showed decreasing transcript expression in the faba bean embryo during seed development. A decreasing expression during embryo development was also observed for transcripts annotated as enzymes catalyzing a majority of the steps from acyl-CoA synthesis to TAG formation, and for transcripts of importance for the breakdown of TAGs to fatty acids and glycerol. The degradation of fatty acids in the peroxisomes, the beta-oxidation cycle, helps plants to recycle co-factors such as NADH or serves as a link to gluconeogenesis. For transcripts important for the beta-oxidation cycle, we observed inconsistent results for the two varieties but with a tendency towards the enrichment of transcripts in the later stages of embryo development. Surprisingly, the expression of transcripts annotated as encoding plant oil body proteins, oleosins, was on an opposite trajectory (as compared to the observed decreasing trend for fatty acid and TAG synthesis), being highly expressed in the later developmental stages of the embryo.

## 4 Discussion

### 4.1 More complex developmental switches in the embryo than in the endosperm

The seed development of faba bean is coordinated by interactions of the seed coat, endosperm, and embryo tissues, and is mainly driven by genetic programs, environmental factors, hormones, and the transport and uptake of photoassimilate sugars. Our distinction into four developmental stages based on visual assessment of horizontally dissected seeds, used throughout this paper, indicated major changes throughout the seeds’ development and maturation with regards to the increased proportion of embryo and the concomitantly decreased proportion of endosperm, as well as the increasing dry matter content. This could be confirmed by significant differences during seed development in both nutrient levels and levels of DETs annotated to encode many important metabolic and developmental markers. In the early seed developmental stages of faba bean, nutrients are stored in the endosperm and from there distributed to the developing embryo (Patrick and Stoddard, 2010). The endosperm tissue is therefore clearly visible at this stage, forming a jelly-like layer between the embryo and seed coat. The faba bean is an exalbuminous seed, and after the replenishment of the endosperm, its function as transient nutrient storage is replaced by the inner seed coat (Patrick and Stoddard, 2010). During this process, the embryo grows fast and finally takes up most of the seed space. The early developmental stages I and II in our study had separable embryo and endosperm tissues and were both phases of nutrient synthesis and accumulation. Stage III and IV instead showed a depleted endosperm and were phases of seed filling, nutrient storage, and desiccation. The larger embryo size in var. Fanfare as compared to Taifun during later developmental stages aligns with the available data on thousand grain weight of mature seeds obtained from variety testing in field (Halling & Hagman, 2020).

The embryo tissue showed a higher number of DETs during seed development belonging to TFs than the endosperm tissue, which is indicative of more complex developmental and metabolic switches in the embryo as compared to the endosperm. This in turn, was in line with the different patterns of storage compound accumulation during seed development observed between tissues in our study, with the levels of starch and oil increasing in the embryo, but in principle staying at a constant level in the endosperm. It was interesting to note that the levels of protein, as determined from total nitrogen, were kept at high and relatively constant levels in both tissues during the first three stages of seed development. The two var. Taifun and Fanfare showed similar spatiotemporal patterns in embryo and endosperm of both storage compound accumulation and of DETs involved in seed development and storage compound biosynthesis, and the varieties could therefore serve as confirmative biological replicates. The variety difference in tannin content highlighted a possible anatomical border between the endosperm and the seed coat based on our light microscopy analysis of fixed seed tissues, with clearly visible dark-brown compartments in the outer cell layers of non-stained slices of seeds of Fanfare, which were absent in seeds of the white-flowering var. Taifun. Although not previously described in faba bean, studies in *Arabidopsis thaliana* are indicative of similar structures containing tannins (Haughn and Chaudhury, 2005; Demonsais et al., 2020).

### 4.2 LAFL-network is regulating early embryo development

Nearly 7% of the genes in higher plants encode TFs, which are proteins with a specific DNA-binding domain that is regulating transcription (Le et al., 2011). Many fundamental parts of plant development are regulated through the activation and repression of target genes by TFs (Verdier et al., 2008). From the analysis of RNAseq data, we could note a similar proportion of TFs (6%) in the list of differently expressed transcripts during the faba bean seed development. Among those, we identified several transcripts annotated as encoding TFs that have been characterized as important in *Arabidopsis thaliana* seed development. The most common occurring transcripts among the identified DETs annotated as TFs in faba bean endosperm and embryo development, all belonged to the biggest TF families in plants and are associated with seed development in several other plant species. For example, we found that WRKY was the most abundant TF-family differentially expressed in both endosperm and embryo tissue during the faba bean seed development. From previous studies on several different plant species, including closely related *Medicago truncatula*, the LAFL network is found to be an important set of TFs that act as master regulators in seed development and the deposition of storage reserves such as starch, lipids, and SSPs (Kagaya et al., 2005; Lalanne et al., 2021). However, very little is known about the LAFL network in faba bean. Our results showed that transcripts homologous to three out of the four TFs part of the network were present in the developing embryo tissue of the faba bean. LEC1 showed decreasing expression during embryo development, ABI3 was increasing and FUS3 remained constant. Interestingly, transcripts homologous to LEC2 were neither found in our dataset nor in the predicted open reading frames of the *Vicia faba* reference genome Hedin/2 (Jayakodi et al., 2023). The trends seen in our data follow prior suggestions that LEC1 is a pioneering TF and primarily functions during early seed development stages (embryogenesis), whereas FUS3 and ABI3 activate maturation-specific processes and thus are mostly involved in later stages of seed development (Schneider et al., 2016). The TFs VAL1 and ASIL1, in turn, act as a repressor of the LAFL network (Jia et al., 2014). This is in accordance with our data where the transcripts homologous to VAL1 and ASIL1 were expressed in an opposite pattern to that of LEC1 in the faba bean embryo during seed development. Future studies of individual genes within these sets are of interest to validate our findings and gain a better understanding of their specific roles in seed development, for example using techniques such as quantitative real-time PCR.

### 4.3 Oil bodies as a putative temporal energy reserve in embryonic tissue

Faba bean is distinguished by a combination of its relatively high protein content (30%) and low-fat content (3%) in mature seeds, in comparison with many other grain legumes (Song et al., 2017). In the chloroplasts and other plastids of the plant cell, fatty acids are *de novo* synthesized from acetyl–CoA to be further distributed to other cell compartments where they can be used for the synthesis of different glycerolipids. Glycerolipids can act as structural components of the cell (membrane lipids) and can serve as energy storage in the form of TAGs (Baud and Lepiniec, 2010). FFAs and glycerol can be converted into TAG as part of the process of lipid metabolism and conversely, when energy is needed, TAG can be digested into FFA and glycerol through lipolysis. Our nutrient analysis revealed that, even though being at a low concentration as compared to other major storage compounds, the level of TAGs (oil) was increasing substantially throughout the seeds’ development, with doubled concentration from stage I to stage III in embryo tissue and a five times higher concentration in stage IV. FFA levels, on the other hand, were low and decreasing during embryo development, possibly for use in TAG biosynthesis or as part of the structural formation of the cells’ membrane formations. The transcription factor WRI1 is known to induce oil synthesis during seed embryo maturation by activating the transcription of genes in glycolysis and *de novo* fatty acid synthesis in *Arabidopsis thaliana*, as well as in several other species, including soybean (Focks and Benning, 1998; Ma et al., 2013; Chen et al., 2018). At an early embryo developmental stage, our transcriptomic data of faba bean showed a high expression of WRI1, as well as of its known target genes in fatty acid synthesis and the earlier parts of plastidic glycolysis (which feeds fatty acid synthesis with carbon precursors), indicating a positive regulation of metabolism for the production of FFAs to be used for TAGs (Grimberg et al., 2015). The same trend of high expression levels early and lower expression levels later during seed development was seen for transcripts encoding enzymes in glycerolipid metabolism that leads to TAG synthesis. Oleosomes, also called lipid droplets or oil bodies, are organelles for the storage of TAG. A matrix of accumulated TAGs is surrounded by a protective phospholipid monolayer, including oil body proteins (Miray et al., 2021). The transcripts encoding oil body proteins showed increased levels as the faba bean embryo developed, which is indicative of increasing oil accumulation. Accordingly, the transcripts for ABI3, a TF known in other plant species to regulate the expression of genes encoding oleosins (Lalanne et al., 2021), was also showing increasing levels during embryo development. The observed increase of TAG levels as well as transcripts encoding for oil body proteins and ABI3, in combination with the opposite pattern of the transcripts encoding for WRI1 and fatty acid biosynthesis, show similarities to the Arabidopsis seed (Ruuska et al., 2002). A decreasing trend of *WRI1* expression during seed development has also been reported in several oil-seed species (Troncoso-Ponce et al., 2011). Intriguingly, despite these similarities to faba bean, the levels of oil in mature seeds of Arabidopsis are much higher. However, the onset of lipid droplet organizing proteins, such as oleosins, have a much higher degree of gene expression overlap with *WRI1* in oil seeds during development. It could therefore be possible that lipids, in the absence of organization as TAG oil bodies by lipid droplet proteins, act as temporal energy storage in faba bean seeds and are turned over during development. However, further biochemical characterization is needed to support such hypothesis.

### 4.4 Proteins are accumulated not only in the embryo but also in the endosperm tissue

Although structurally different among different plant species, seed storage proteins (SSPs) share the characteristics of accumulating to high levels in specific seed-organ tissues at certain time points of the seed development (Krishnan and Coe, 2001) and are mainly regulated at the transcriptional level (Verdier et al., 2008). Our study showed that the total protein content, based on total nitrogen determination, was high in the embryo tissue already at an early stage of development (40% by dry weight) and then decreased slightly towards seed maturation (31-33% by dry weight) and that the level was even slightly higher in endosperm tissue. The seed protein content in both varieties were 28-29% as determined from variety tests in field (Halling and Hagman, 2020), which is just below the content in embryo tissue at our latest developmental stage analyzed. A higher seed protein content in the early developing stages as compared to later stages has previously been observed in other investigations of faba bean seeds (Warsame et al., 2022) as well as in other legume seeds (Sital et al., 2011; Zhang et al., 2021). However, it should be noted that total protein levels, estimated through total nitrogen determinations, do not exclusively represent storage proteins. This is because a portion of the total nitrogen comes from structural proteins, enzymes, amides, free amino acids etc. (Ezeagu et al., 2002). This nitrogen portion can be expected to be more prominent during the early stages of seed development, characterized by rapid cell division, as compared to later stages when there is a substantial accumulation of storage proteins. Nevertheless, light green histochemical staining, which stains proteins by binding to free basic side chains of a protein by its sulfonic acid group (Oud et al., 1984), of fixed and sliced seed tissues confirmed the presence of high protein levels in the early phases of both embryo and endosperm development.

The main storage proteins in faba bean are globulins, which are comprised of legumin and vicilin/convicilin, accounting for almost half and one-third of the SSPs respectively (Warsame et al., 2020). They are known to be synthesized at 21-28 DaF in faba bean (Patrick and Stoddard, 2010), time points that within this study match phases II and III of seed development. The transcriptomic data showed an increasing expression for transcripts annotated as SSPs during embryo development, which is in line with an increasing protein content in absolute terms (mg protein/seed).

### 4.5 Starch is accumulating in the later stages of embryo maturation

Starch makes out the major part of the carbohydrates found in the mature faba bean seed, with levels between 30%–42% of dw (Cerning and Guilbot, 1975). During the day, photosynthates are exported from the chloroplast and partitioned as sucrose in the phloem, either for immediate uses (growth assimilates), temporary storage as transient starch in the cytosol, or imported to amyloplasts for long-term energy storage, so-called storage starch (MacNeill et al., 2017). With the help of histochemical staining, we could visually show that the seed coat at early seed developmental stages is rich in amyloplasts, organelles that synthesize and store starch, later on providing a possible carbon source for the maturing embryo (Weber et al., 2005). Our nutrient analysis showed that the embryo, in turn, accumulates starch increasingly towards the later developmental stages which could further be confirmed by our transcriptome results showing an increasing trend of transcripts levels encoding enzymes in the cytosolic glycolytic pathway. As seed size stabilizes and the endosperm is depleted, the cytosolic glycolytic activity increases to support starch and protein storage. In glycolysis, plants oxidize hexoses to generate ATP and organic acids and produce building blocks for anabolism. This is in agreement with our nutrient analysis which showed the highest levels of starch content in the later stages (III and IV) of seed development, as well as with our increasing transcriptomic data of homologous genes annotated to encode starch synthase, 1,4-alpha-glucan branching enzyme, and ADP-glucose pyrophosphorylase, all playing important roles in the starch biosynthesis pathway in plants (Stitt and Zeeman, 2012).

### 4.6 Conclusion and future directions

Seed quality is an important breeding target in faba bean to nourish an increasing population with better and more sustainably produced food and animal feed. The complex relationship between seed development, nutrient biosynthesis, and the resulting seed quality of faba bean is therefore an important area of study that can be of interest to the whole value chain of faba bean, from plant breeding to food and feed producers and consumers. Increased knowledge of the genetic regulation of synthesis and storage patterns of protein, starch, and oil of seeds is an important step towards identifying specific markers that can be used in efficient breeding of protein-rich legumes. In this study, we identified highly differentially expressed transcripts during seed development in faba bean that were annotated as encoding enzymes in the synthetic pathways for these storage compounds, as well as TFs known to regulate embryo development. This can form the basis for further research to identify breeding targets for desired seed qualities in faba bean. One interesting suggested breeding target is the subunit composition of storage proteins towards a higher legumin:vicilin ratio which could enhance the faba bean’s nutritional qualities significantly with regards to amino acid composition (Warsame et al., 2020). A somewhat surprising finding of the current study of faba bean was that the expression pattern of genes involved in fatty acid and oil biosynthesis was similar to that in high-oil accumulating plant species. Therefore, another interesting breeding target could be to explore possibilities of increasing the oil content from the current very low level in faba bean, having in mind that soybean is today a dual-purpose legume crop with a high economic value for both oil and protein (Guo et al., 2022). In starch-rich grain legume crops, such as faba bean, there is usually a trade-off towards oil. Varieties with lower starch content could therefore show a higher capacity to store lipids, a useful trait for further biotechnological modifications toward increased oil content (Song et al., 2017).

## Data availability statement

The datasets for this study can be found in the Sequence Read Archive (SRA) database at NCBI under BioProject accession number PRJNA861904. https://www.ncbi.nlm.nih.gov/bioproject/PRJNA861904.

## Author contributions

HO: Data curation, Formal analysis, Investigation, Methodology, Validation, Visualization, Writing – original draft. GS: Data curation, Formal analysis, Methodology, Software, Writing – review & editing. PH: Conceptualization, Funding acquisition, Methodology, Resources, Supervision, Validation, Writing – review & editing. ÅG: Conceptualization, Funding acquisition, Methodology, Project administration, Resources, Supervision, Validation, Writing – review & editing.

## Supplementary material

SUPPLEMENTARY MATERIAL
**Supplementary Figures 1–2** and **Supplementary Table 1–5**.

SUPPLEMENTARY DATASHEET 1 List of all differentially expressed transcripts from all pairwise genotype comparisons.

SUPPLEMENTARY DATASHEET 2 AT Locus names and search queries for transcripts used in **Figure 7A**.
