# Supplementary material for: Spatio-temporal transcriptome and storage compound profiles of developing faba bean (Vicia faba) seed tissues
Source: Front Plant Sci. 2024 Feb 6;15:1284997. doi: 10.3389/fpls.2024.1284997 (PMC10877042; doi:10.3389/fpls.2024.1284997)
Supplement: SUPPLEMENTARY MATERIAL — Supplementary Figures 1–2 and Supplementary Table 1–5 . [file Presentation_1.pdf]

Supplementary material

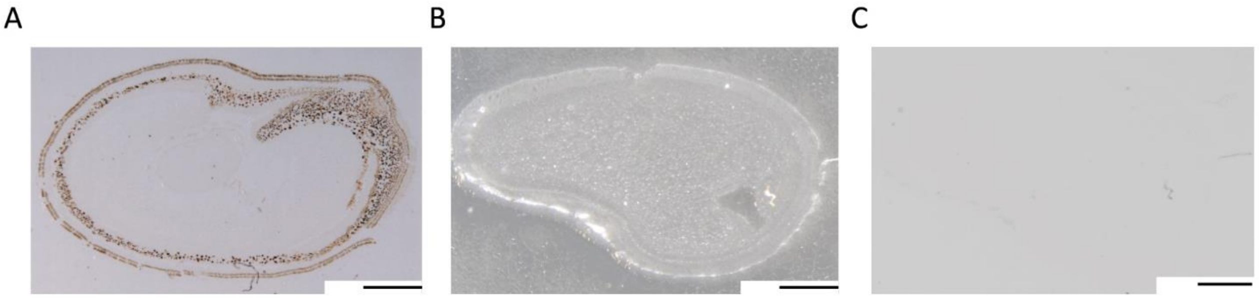

Supplementary Figure 1. Light microscopy picture of non-stained sections of paraffin-embedded seeds. Pictures are showing the varieties Fanfare **(A)** and Taifun **(B)** indicating the presence of tannins in dark-brown color in Fanfare at developmental stage I-II. Dark field setting was used for the microscope for var. Taifun, since otherwise no structures could be seen at all **(C)**. Scale bar: 1mm.

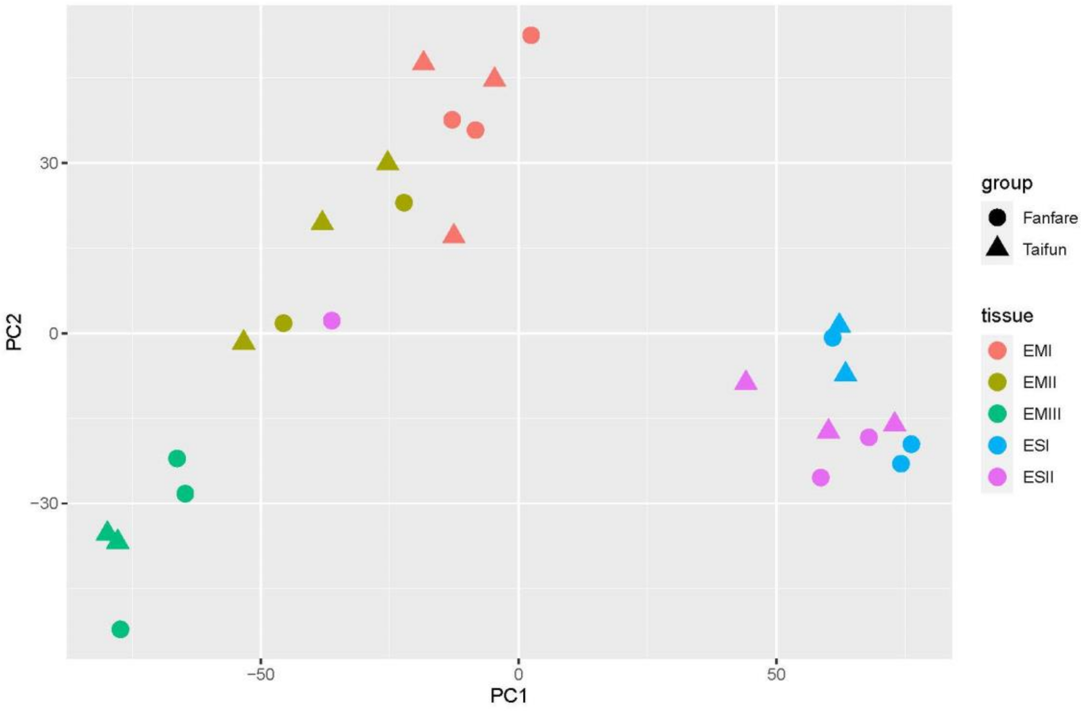

Supplementary Figure 2. Principal component analysis (PCA) plot of the read count data for all biological replicates used in the study; embryo (EM) at developmental stages I to III and endosperm (ES) at stages I and II, for var. Fanfare and Taifun respectively.

Supplementary Table 1. Number of biological replicates for varieties Fanfare and Taifun used for RNA extraction.

	**Fanfare**	**Taifun**
**EMI**	3	3
**EMII**	2	3
**EMIII**	3	2
**ESI**	3	2
**ESII**	3	3
**PII**	1	1
**PS**	3	3

Supplementary Table 2. The sequence reads are shown in Millions (Mseq) before and after filtering, sample IDs, sample short and full names described here were used for the de-novo transcriptome assembly, in the differential expression analysis and the SRA raw data repository.

SAMPLEID			MSEQ	
**ID1**	**ID2**	**SampleID_SRA**	**Before filter**	**After filter**
Fanfare	EMI	F1_EMI_lib435225	83.6	77.2
Fanfare	EMI	F2_EMI_lib435226	75.6	70.6
Fanfare	EMI	F6_EMI_lib435227	87.4	78.6
Fanfare	EMII	F1_EMII_lib435231	58.8	53
Fanfare	EMII	F5_EMII_lib435232	103.2	97.2
Fanfare	EMIII	F1_EMIII_lib435236	82.2	72.8
Fanfare	EMIII	F3_EMIII_lib435237	69.4	65.6
Fanfare	EMIII	F5_EMIII_lib435238	63.6	59.2
Fanfare	ESI	F1_ESI_lib435241	79.2	70.2
Fanfare	ESI	F2_ESI_lib435242	89.2	67.6
Fanfare	ESI	F6_ESI_lib435243	66.8	56.4
Fanfare	ESII	F1_ESII_lib435246	76.6	70.2
Fanfare	ESII	F2_ESII_lib435247	86.8	64
Fanfare	ESII	F3_ESII_lib435248	89.8	73.8
Taifun	EMI	T3_EMI_lib435228	89.2	84
Taifun	EMI	T4_EMI_lib435229	78.4	73.6
Taifun	EMI	T5_EMI_lib435230	74.6	70
Taifun	EMII	T4_EMII_lib441948	68	62.8
Taifun	EMII	T5_EMII_lib435234	70.4	66.2
Taifun	EMII	T6_EMII_lib435235	67.8	64.2
Taifun	EMIII	T5_EMIII_lib435239	77.2	72.4
Taifun	EMIII	T8_EMIII_lib435240	72.4	67.6
Taifun	ESI	T5_ESI_lib435244	69.6	64.6
Taifun	ESI	T6_ESI_lib435245	67.8	51.8

Taifun	ESII	T4_ESII_lib435249	66.2	62.4
Taifun	ESII	T5_ESII_lib435250	67.6	62.8
Taifun	ESII	T6_ESII_lib435251	81.6	76.2

Supplementary Table 3. A total of 227,336 predicted genes from the de-novo faba bean transcriptome assembly (based on the embryo, endosperm, pericarp, petals, and sepals tissues) were blasted against four organism's transcripts (unique target gene hit numbers are in brackets). Transcription factor (TF) databases blast annotations were based on three model organisms.

**Annotation**	***M. truncatula***	***P. sativum***	***G. max***	***A. thaliana***
All de-novo transcripts (unique target gene hits)	80438 (24098)	72102 (26779)	76030 (28897)	52633 (19169)
De-novo transcripts with ORFs [one or more ORFs per transcript] (unique target gene hits)	42272 (21198)	39716 (22084)	41639 (23716)	35382 (16826)
Unique de-novo transcripts with ORFs (unique target gene hits)	30005 (21198)	27966 (22084)	29559 (23716)	25354 (16826)
PlantTFDB (unique target gene hits; TF taxonomic groups)	5764 (1673; 57)	-	3737 (1978; 57)	4269 (1132; 56)
iTak (unique target gene hits; TF taxonomic groups)	8522 (2230; 92)	-	5657 (1541; 92)	5827 (2732; 92)

Supplementary Table 4. All pairwise comparisons between the genotypes, by using minimum quality criteria, resulted in an average of 21,089 differentially expressed transcripts (DETs). However, by setting more stringent thresholds (one adjusted value of FDR < 0.05, and another adjusted value of both FDR < 0.05 and Log2FoldChange (Log2FC) > 1.0), the number of DETs in the pairwise comparisons were much reduced, and only the most stringent of the two thresholds was used for further analysis.

	Fanfare				Taifun			
**Comparisons**	**ESI vs. ESII**	**EMI vs. EMII**	**EMII vs. EMIII**	**EMI vs. EMIII**	**ESI vs. ESII**	**EMI vs. EMII**	**EMII vs. EMIII**	**EMI vs. EMIII**
Total DE transcripts	20875	20537	19349	21030	22943	22261	20466	21249
Number of DETs ($p5e^{-2}$_c0)	135	1726	1416	7456	935	1489	5834	9725
Number of DETs ($p5e^{-2}$ _c1)	**125**	**884**	**1220**	**5715**	**899**	**910**	**3842**	**6839**

Supplementary Table 5. The most common differentially expressed transcripts encoding transcription factors in developing embryo and endosperm tissue. Tables show the number of DETs during embryo development **(A)** and endosperm development **(B)**.

A		B	
**Transcription factor family name**	**No DETs in Developing Embryo**	**Transcription factor family name**	**No DETs in Developing Endosperm**
WRKY	199	WRKY	9
NAC	112	bHLH, NAC	6
bHLH, C2H2	29	HD-ZIP, C2H2	4
bZIP	26	ERF	3
C3H	23	MYB, HSF, M-type_MADS, Dof,	2
MYB, HD-ZIP, GRAS	16	Trihelix, NF-YB, MIKC_MADS, LSD, LBD, G2-like, C3H, BES1, ARF, ZF-HD, SBP, MYB_related, GATA, CO-like, B3, ARR-B, AP2	1
MYB_related, Trihelix, ERF	14		
ARF	12		
GATA, Dof, CO-like, B3	11		
AP2	10		
HSF, G2-like	9		
TALE, NF-YB, ARR-B	7		
ZF-HD, CAMTA	6		
TCP, NF-YA, GRF, M-type_MADS, FAR1	5		
WOX, Nin-like, YABBY	4		
NF-YC, MIKC_MADS, SRS, E2F/DP, BES1	3		

Whirly, SBP, LBD, HB-PHD, EIL, DBB, CPP, NF-X1, HB-other	2	
SAP, GeBP, BBR-BPC, LFY	1	
